# Whole-exome and whole-transcriptome sequencing of canine mammary gland tumors

**DOI:** 10.1038/s41597-019-0149-8

**Published:** 2019-08-14

**Authors:** Ka-Kyung Kim, Byung-Joon Seung, Dohyun Kim, Hee-Myung Park, Sejoon Lee, Doo-Won Song, Gunho Lee, Jae-Ho Cheong, Hojung Nam, Jung-Hyang Sur, Sangwoo Kim

**Affiliations:** 1 Department of Biomedical Systems Informatics and Brain Korea 21 PLUS Project for Medical Science, Yonsei University College of Medicine, Seoul, 03722 Republic of Korea; 2 Department of Veterinary Pathology, Small Animal Tumor Diagnostic Center, College of Veterinary Medicine, Konkuk University, Seoul, 05029 Republic of Korea; 3 School of Electrical Engineering and Computer Science, Gwangju Institute of Science and Technology (GIST), Gwangju, 61005 Republic of Korea; 4 Department of Veterinary Internal Medicine, College of Veterinary Medicine, Konkuk University, Seoul, 05029 Republic of Korea; 5 Precision Medicine Center, Seoul National University Bundang Hospital, Seongnam, 13620 Republic of Korea; 6 Graduate Program for Nanomedical Science, Yonsei University, Seoul, 03722 Republic of Korea; 7 Department of Surgery, Severance Hospital, Yonsei University College of Medicine, Seoul, 03722 Republic of Korea

**Keywords:** Cancer genomics, Cancer genetics, Next-generation sequencing

## Abstract

Studies of naturally occurring cancers in dogs, which share many genetic and environmental factors with humans, provide valuable information as a comparative model for studying the mechanisms of human cancer pathogenesis. While individual and small-scale studies of canine cancers are underway, more generalized multi-omics studies have not been attempted due to the lack of large-scale and well-controlled genomic data. Here, we produced reliable whole-exome and whole-transcriptome sequencing data of 197 canine mammary cancers and their matched controls, annotated with rich clinical and biological features. Our dataset provides useful reference points for comparative analysis with human cancers and for developing novel diagnostic and therapeutic technologies for cancers in pet dogs.

## Abstract (summary)

**Table:** 

Design Type(s)	disease state design • transcription profiling design • parallel group design
Measurement Type(s)	transcription profiling assay • exome
Technology Type(s)	RNA sequencing • DNA sequencing
Factor Type(s)	breed • age • experimental condition • diagnosis
Sample Characteristic(s)	Canis lupus familiaris • tissue

Machine-accessible metadata file describing the reported data (ISA-Tab format)

## Background & Summary

High-quality sequencing has clarified the dog genome with a coverage of >99%^1,2^. Moreover, the availability of a high-coverage reference genome and the emergence of higher-resolution next-generation sequencing have led to the identification of genomic structures coded by the dog genome, as provided in CanFam3.1^3^. Based on such genomic information, cost-efficient next-generation sequencing has become available, allowing researchers to target specific coding regions and other regulatory elements for dogs. We suspect that whole-exome sequencing (WES) and whole-transcriptome sequencing (WTS) could be applied to discover single nucleotide variations (SNVs)^4,5^ and mutations causing diseases in dogs, such as in progressive retinal atrophy^6^.

Spontaneously occurring canine mammary gland tumors (CMTs) are of great interest to cancer researchers due to their clinical importance. CMTs are the most prevalent neoplasm in intact female dogs, and approximately 50% are malignant^7,8^. Interestingly, CMTs have been found to be promising cancer models with which to study human breast cancer due to their marked biological and clinical similarities^9,10^. Indeed, histopathological classification and histological grading of CMTs have been adopted from those of human breast cancer^11–13^ and, just as in humans, are actively used as prognostic indicators^11,12,14^. A recent multi-omics study of CMTs from 12 individual dogs characterized genomic features of two subtypes (simple carcinomas and complex carcinomas) and identified similarities and differences therein with human breast cancer^15^. To establish a better model and a more accurate profile of the molecular landscape of CMT, well-controlled multi-omics data for a larger cohort is desired.

**Fig. 1:**
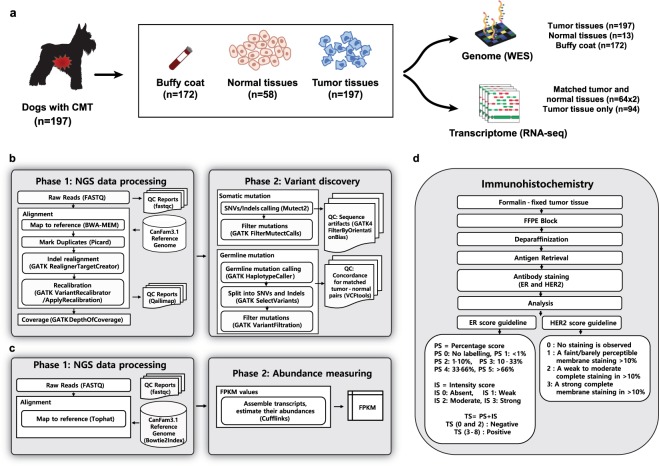
Overview of study design and workflow. (**a**) Study design of WES and RNA-Seq analysis. (**b**) Workflow of the WES data processing and variant calling. (**c**) Workflow of the RNA-Seq data processing. (**d**) Immunohistochemistry (IHC) workflow.

Accordingly, to provide a useful resource for genomic analysis, we produced WES and WTS sequencing data from 197 and 158 dogs with CMTs, respectively. Among them, 185 of 197 of the WES (DNA-Seq) and 64 of the 158 WTS (RNA-Seq) specimens were matched with appropriate controls: buffy coats or normal mammary tissue for WES and normal mammary tissue for WTS were matched. Histopathological characteristics were evaluated in all tumor samples, including histopathological subtype, grade, and lymphatic invasion, and samples were annotated with corresponding sequencing data. In addition, immunohistochemical evaluation was performed in 189 samples to determine estrogen receptor (ER) and human epidermal growth factor receptor 2 (HER2) status. The raw sequencing data were aligned to the CanFam3.1 reference genome following the Best Practices announced by the Genome Analysis Toolkit (GATK, see Methods)^16^. We performed multiple quality control processes to confirm the quality of the sequencing and matched pairs of buffy coats and tumor tissues. Finally, the raw and aligned sequencing data, as well as normalized gene expression values (FPKM), were deposited in a public repository, along with the recorded clinical and biological information. A visual summary of the study design and workflow is shown in Fig. 1.

Overall, we are sharing a complete WES and WTS dataset that is ready for further biological analysis. We anticipate that this resource can be utilized for devising and validating various hypotheses in studies of comparative oncology between canine and human cancers.

## Methods

### Sample collection and preparation

Fresh and formalin-fixed paraffin-embedded (FFPE) tumor tissue samples of spontaneously occurring canine mammary tumors, adjacent normal tissue samples, and blood samples were obtained from privately-owned pet dogs via private veterinary clinics with informed consent the owners. Tissue samples were obtained as a part of routine diagnostic procedures, and blood samples were collected for research following the guidelines of and approval from the Institutional Animal Care and Use Committee of Konkuk University (KU16106 and KU17162). Fresh tissue samples were immediately transferred to RNAlater™ (Thermo Fisher Scientific, Vilnius, Lithuania), refrigerated overnight at 4 °C, and then stored at −80 °C until ready for analysis. For histopathology and immunohistochemistry (IHC) analysis, tissue samples were fixed in 10% neutral buffered formalin, processed routinely and embedded in paraffin wax. Blood samples were centrifuged, and buffy coats were isolated and stored at −80 °C until required for DNA extraction.

Genomic DNA was extracted from tissues using QIAamp DNA mini kits (Qiagen, Germany), and total RNA was extracted from tissues using RNeasy mini kits (Qiagen). Buffy coat DNA was extracted using QIAamp DNA blood mini kits (Qiagen) according to the manufacturer’s instructions.

### Histopathology

Sections (4-μm thick) from the FFPE blocks were stained with hematoxylin and eosin and were diagnosed by veterinary pathologists (B.J.S. and J.H.S.). Histological subtype was determined by the World Health Organization classification^11^. Histological grade was assessed according to Peña system^17^, exclusively on the neoplastic epithelial component. In the case of mammary osteosarcoma and mammary fibrosarcoma, histological grade was assessed according to the grading system for canine osteosarcoma^18^ and the grading system for cutaneous and subcutaneous soft tissue sarcoma in dogs^19^, respectively. Lymphatic invasion, defined as infiltration of tumor cells in peritumoral lymphatic vessels (all cases) or infiltration of regional lymph nodes (only available cases), was also assessed.

### Immunohistochemistry

Formalin-fixed paraffin-embedded canine mammary tumor samples (except osteosarcoma, fibrosarcoma, and poorly fixed tissues) underwent detection of estrogen receptor (ER) and human epidermal growth factor receptor 2 (HER2) by IHC with primary antibodies for ER (Biogenex, San Ramon, CA, USA) and HER2 (Dako, Glostrup, Denmark). Immunohistochemistry was performed as described in the previous publication^20^. Adjacent normal mammary gland or mammary hyperplasia were used as positive controls for ER antibody. Control slides known to be positive for HER2 were used as controls for HER2 antibody. Isotype-matched antibodies were used as negative controls.

ER and HER2 status were evaluated by the two veterinary pathologists mentioned above. Only epithelial tumor cells of representative areas were evaluated. Expression of ER was evaluated based on guidelines suggested by Pena *et al*.^17^. Expression of HER2 was measured based on recent guidelines recommended by the American Society of Clinical Oncology/College of American Pathologists^21^. Due to observation of non-specific cytoplasmic staining (according to human criteria) in canine tissues, as described by Burrai *et al*.^22^, only membrane stains were considered for scoring in this study.

### Whole-exome sequencing

We sequenced 197 samples following the Illumina HiSeq 2500 protocol outsourced to Theragenetex. Two hundred nanograms of fragmented DNA was prepared to construct libraries with the SureSelect Canine All Exon Kit (Agilent, Inc., USA) using the manufacturer’s protocol. Briefly, qualified genomic DNA samples were randomly fragmented by Covaris, followed by adapter ligation, purification, hybridization, and PCR. Captured libraries were subjected to an Agilent 2100 Bioanalyzer to evaluate quality and were loaded on to the Illumina HiSeq sequencer, according to the manufacturer’s recommendations.

### RNA sequencing

Before library construction, RNA 6000 Nano kits (Agilent Technologies, CA) were used to assess RNA quality. For cDNA library construction, 1 ug of RNA was obtained and purified with oligo-dT magnetic beads. Fragmentation was performed with purified mRNA, and double-stranded cDNAs were synthesized. The cDNAs were primed with poly-A, and sequencing adapters were connected using TruSeq RNA sample prep kits (Illumina, CA). Fragments were filtered to a specific length using BluePippin 2% agarose gel cassettes (Sage Science, MA), and PCR amplification was conducted. Fragment lengths and quality were electrophoretically verified with Agilent High Sensitivity DNA kits (Agilent Technologies, CA). Libraries were observed with a window spanning an average of 392 bp, standard deviation of 66. Finally, Illumina HiSeq 2500 was used for sequencing (Illumina, CA).

### Processing of whole-exome sequencing data

Sequences were aligned to the CanFam3.1 reference genome with BWA-MEM2^23^ and were output in a technology-independent SAM/BAM reference file format. Next, duplicate fragments were marked and eliminated with Picard (version 2.2) (http://picard.sourceforge.net). After assessing mapping quality and filtering out low-quality mapped reads, paired read information was evaluated to ensure that all mate-pair information was in sync between each read. Then, processes of removing PCR duplicates, indel realignment, fixing mate information, base quality score recalibration, and variant quality score recalibration on putative SNVs and indels were performed.

Germline and somatic mutations were called from the alignment files using GATK4.0^24^ following GATK Best Practices recommendations, with using CanFam3.1 (Ensembl Release 91) as reference: the whole pipeline was implemented in-house (see Code Availability). The VCF file produced by the pipeline uses reference bases on the positive strand of CanFam3.1 in the REF field, and variants are shown in the ALT field. We calculated the depth of coverage using GATK and then followed the typical XHMM workflow for CNV calls^25^.

### Processing of RNA sequencing data

RNA-Seq data of 158 tumor samples and 64 normal samples matched with tumors were sequenced. Prepared reads (FASTQ files) were mapped to the canine reference genome CanFam3.1 (Canis lupus familiaris) using TopHat (v.2.0.9), with Ensembl gene annotation and fr-firststrand library type. FPKM (Fragments Per Kilobase of Transcript per Million) values were calculated by Cufflinks (v2.1.1) using aligned bam files.

## Data Records

The raw FASTQ files of the WES and RNA-Seq data produced by Illumina Highseq. 2500 are available from the Sequence Read Archive^26,27^. The RAW FASTQ files and FPKM values of RNA-Seq are available in the Gene Expression Omnibus database^28^. All steps used to process the raw files in order to create the final file are available at our GitHub repository (see Code availability). Sample characteristics are summarized in Online-only Table 1. Details on age, neuter status, histopathology descriptions, and immunohistochemical evaluation are deposited at Figshare^29^. Additional metadata links to SRA and GEO with clinical information are provide at Figshare^29^. The VCF files for germline mutations (SNPs and indels) of 197 CMTs and 185 normal samples called by GATK haplotype caller and for somatic variant calls of whole exome sequencing of 185 matched CMT and normal samples called by Mutect2 can be accessed at Figshare^29^.

## Technical Validation

### Quality validation

**Fig. 2:**
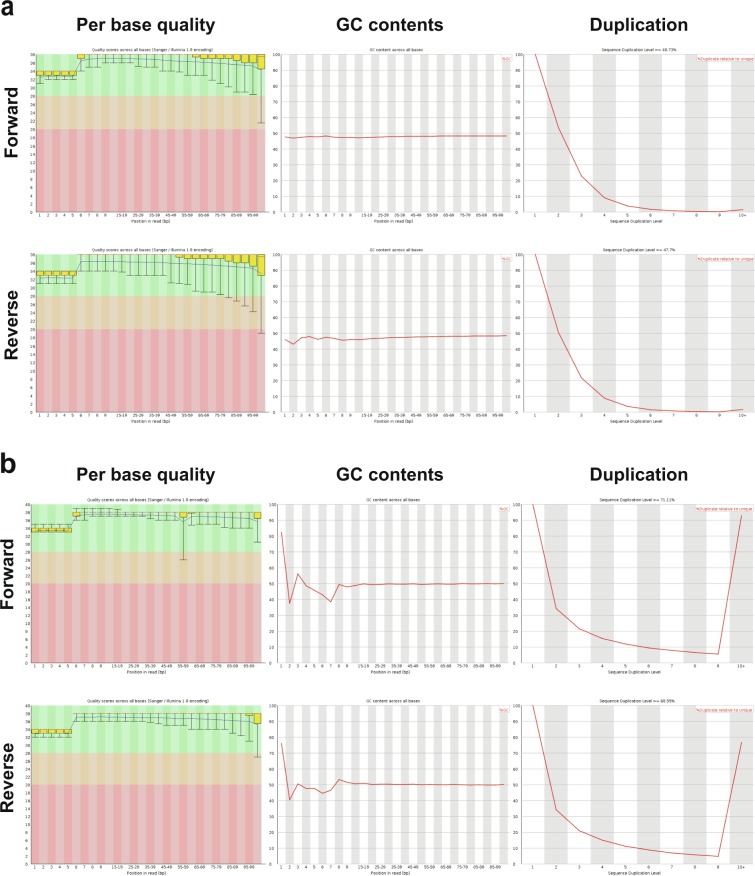
Confirmation of sequencing quality. (**a**) Quality score, GC content across all bases, and sequence duplication level in CMT-193 tumor (WES). (**b**) Quality score, GC content across all bases, and sequence duplication level in CMT-193 tumor (RNA-Seq).

**Fig. 3:**
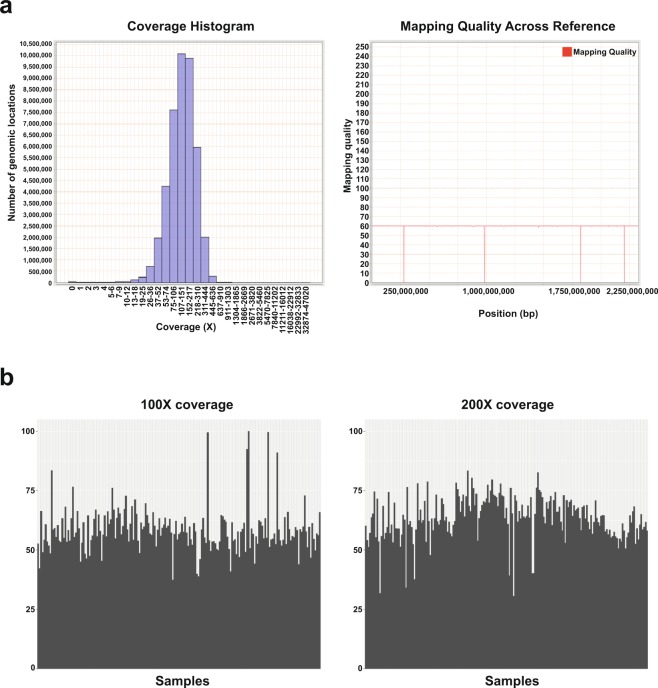
Confirmation of sequencing quality and coverage. (**a**) Mapping quality and coverage in CMT-193 tumor (WES). 100X and 200X coverage in WES. Each bar on the x-axis represents a single sample, and the percentage on the y-axis indicates the percentage of bases out of all sequenced bases.

We validated quality of sequencing following the previous reported QC measures^30^. We used FASTQC v0.10.1 to analyze data quality via several measures, including sequence quality per base, GC content per sequence, sequence duplication levels, and quality score distribution over all sequences in the FASTQ files^29^. We randomly selected sample CMT-193 as a representative sample. Representative summary plots are provided in Fig. 2. High quality scores per base were shown, having a median quality score more than 30 both in WES (Fig. 2a left column) and RNA-Seq (Fig. 2b left column). The average quality score for overall sequences showed high scores above 30. Those score measures indicate that a large amount of the sequences in a run had high quality. The GC content of any strays were less than 5% in WES showing systemic bias free in sequence library (Fig. 2a middle column). The GC contents were uniform mostly although there were some bias in 1~8 bp in RNA-Seq (Fig. 2b). Examining sequence bias during polymerase chain reaction (PCR) amplification, we found that less than 2% of sequences were shown over 10 times in both platforms although RNA-Seq data have higher duplication rate than WES (Fig. 2a,b right column). We analyzed the quality score distribution of all sequences to verify if a subset of sequences had globally good quality. We applied Qualimap v2.2 to examine quality of sequencing alignment data according to features of the mapped reads. Qualimap highlights random errors and systematic biases, including PCR problems, GC content bias, and read contamination^31^. Mean mapping quality was around 60, and mean coverage was around 150X in WES (Fig. 3a). All other FASTQC and Qualimap files were shown to have quality metrics similar to those for randomly selected sample CMT-193. We calculated coverage using the “DepthOfCoverage” function in GATK and then calculated the percentage of bases with at least 100X and 200X coverage (Fig. 3b).

### Concordance and swap for matched tumor–normal pairs

We checked concordance and swap to identify abnormal patterns of samples with large numbers of somatic mutations. We compared germline mutations in all samples pairwise with the following conditions: total allele depth >10, reference allele depth >=90% for genotypes (0/0), reference allele depth >=40%, reference allele depth <60% for genotypes (0/1), and alternative allele depth >=90% for genotypes (1/1). We calculated concordance ratios between all pair samples. Most alleles of tumor-normal pairs with the same sample ID were best matched; however, many abnormal normal samples had higher concordance with other unpaired tumor samples. We compared germline mutations in abnormal samples among the WES with RNA-Seq data and found high concordance between platforms for the same sample IDs. From the analysis, we found 23 unmatched pairs and the possibility of swapping for buffy coats. We excluded them from the downstream analysis. Among unmatched paired samples, we re-sequenced 11 normal samples whose normal tissues were available.

### Sequence artifacts during shearing

Next-generation sequencing can produce sequence context-dependent artifacts, such as oxidation of guanine to 8-oxoguanine (OxoG) and FFPE deamination during genomic library preparation^32^. OxoG artifacts stem from oxidation of guanine to 8-oxoguanine, which results in G to T transversions. FFPE artifacts might be caused by formaldehyde deamination of cytosines, which results in C to T transitions. We ran the GATK4 “FilterByOrientationBias” function on Mutect2 calls and ensured that there was no OxoG or FFPE deamination in our dataset. Additionally, we manually checked whether samples had high C to A versus G to T conversion ratio.

## Usage Notes

The bioinformatics pipeline used on our dataset, as outlined in Fig. 1, was mostly carried out using freely available and open access tools. Additionally, we conducted quality control analyses at multiple steps due to the possibilities of sample swapping and relatively poor standardization of canine analysis pipelines.

Detailed histology descriptions and IHC results of canine mammary tumors are described at Figshare^29^. Despite limitations in molecular classification in dogs and non-specific staining (HER2) in this study, our data will be helpful to further canine mammary tumor studies.

The size and the composition of the deposited dataset are subject to change according to further quality control and additional sequencing. The updated information will be noted in the corresponding data repository sites.

### ISA-Tab metadata file

Download metadata file

## Data Availability

A full description of our analysis pipeline, describing all of the programs and parameters used, is openly available at https://github.com/irobii/cmt. The Markdown file in the pipeline folder documents each step of the pipeline, as well as provides external links to relevant sources for further information.

## Online-only Table

**Online-only Table 1:** Sample characteristics.

Characteristics	No.	%
**Ages**		
<7	8	4.06%
7	6	3.05%
8	10	5.08%
9	9	4.57%
10	23	11.68%
11	15	7.61%
12	30	15.23%
13	26	13.20%
14	32	16.24%
15	8	4.06%
16	12	6.09%
>16	4	2.03%
Not applicable	14	7.11%
**Neuter status**		
Intact	128	64.97%
Neutered	60	30.46%
Not applicable	9	4.57%
**Breed**		
Maltese	61	30.96%
Shih Tzu	24	12.18%
Yorkshire Terrier	20	10.15%
Poodle	17	8.63%
Cocker Spaniel	9	4.57%
Pomeranian	7	3.55%
Schnauzer	7	3.55%
Chihuahua	5	2.54%
Dachshund	5	2.54%
Mixed	20	10.15%
Etc	12	6.09%
Not applicable	10	5.08%
**Diagnosis**		
Benign tumor	57	28.93%
Simple carcinoma, grade 1	35	17.77%
Simple carcinoma, grade 2	14	7.11%
Simple carcinoma, grade 3	26	13.20%
Complex carcinoma, grade 1	28	14.21%
Complex carcinoma, grade 2	9	4.57%
Complex carcinoma, grade 3	3	1.52%
Carcinoma in benign mixed tumor, grade 1	13	6.60%
Carcinoma in benign mixed tumor, grade 2	2	1.02%
Other types of malignant tumor	10	5.08%
**Lymphatic invasion**		
Present	18	9.14%
Absent	179	90.86%
**ER (Allred Score)**		
Positive (3–8)	160	84.66%
Negative (0–2)	29	15.34%
**HER2 (Hercep test Score)**		
0	18	9.52%
1	38	20.11%
2	86	45.50%
3	47	24.87%
